# Postoperative Massive Pulmonary Embolism Due to Superficial Vein Thrombosis of the Upper Limb

**DOI:** 10.14740/jocmr2362w

**Published:** 2016-02-27

**Authors:** Marco Cascella, Daniela Viscardi, Francesca Bifulco, Arturo Cuomo

**Affiliations:** a Division of Anesthesiology, Department of Anesthesiology, Endoscopy and Cardiology, Istituto Nazionale Tumori Fondazione Pascale, Naples, Italy

**Keywords:** Superficial vein, Thrombosis, Upper extremities, Deep venous thrombosis, Pulmonary embolism, Pulseless electrical activity

## Abstract

It is well known that deep vein thrombosis of the upper extremities is linked to high morbidity/mortality, resulting in 12-20% of all documented pulmonary embolism; however, there are few data about thromboembolism originating from a vein and/or a branch of a superficial vein of the upper extremities. Pulmonary embolism secondary to upper limb superficial vein thrombosis (not combined with upper extremities deep vein thrombosis) is a very rare clinical manifestation with few cases reported in the literature. We report a rare case of thrombophlebitis in departure from a superficial branch of the cephalic vein of the right arm, complicated by cardiac arrest secondary to a massive pulmonary embolism in a patient who underwent major surgery for ovarian cancer. We discuss on the numerous thrombotic risk factors, triggering a cascade of reactions and resulting in a potential fatal clinical manifestation.

## Introduction

It is well known that deep vein thrombosis of the upper extremities (UEDVT) is linked to high morbidity/mortality, resulting in 12-20% of all documented pulmonary embolism (PE) [1, 2]; however, there are few data about thromboembolism originating from a vein and/or a branch of a superficial vein of the upper extremities.

The true incidence of superficial vein thrombosis (SVT) is underestimated because many cases remain undiagnosed. Although several cases of PE following an SVT of lower limb are reported [3], PE secondary to upper limb SVT (without UEDVT) is a very rare clinical manifestation with few cases reported in the literature [4, 5].

We report a rare case of thrombophlebitis in departure from a superficial branch of the cephalic vein of the right arm, complicated by cardiac arrest secondary to a massive PE in a patient who underwent major surgery for ovarian cancer.

## Case Report

A Caucasian female patient aged 48 years, with normal body mass index (weight 60 kg), underwent bilateral hysteroannessectomy with omentectomy and lymphadenectomy for ovarian cancer. Standard deep venous thrombosis prophylaxis with low molecular weight heparin (Nadroparine 2850 Anti Xa IU sc) was employed. After the operation, the patient was transferred to the post-anesthesia care unit (PACU). Postoperative course in the first 24 h was normal, without any dyspnea, tachypnea, or hemodynamic changes. PaO_2_/FiO_2_ was normal.

After 24 h, during the mobilization maneuvers for the discharge from PACU, the patient had a sudden loss of consciousness with cardiac arrest at the monitor. We started cardiopulmonary resuscitation (CPR). After 15 min of CPR, the patient had a pulseless electrical activity (PEA). However, at Doppler echocardiography, there was a residual contractile activity. After another 20 min of CPR pulse, consciousness reappeared.

**Figure 1:**
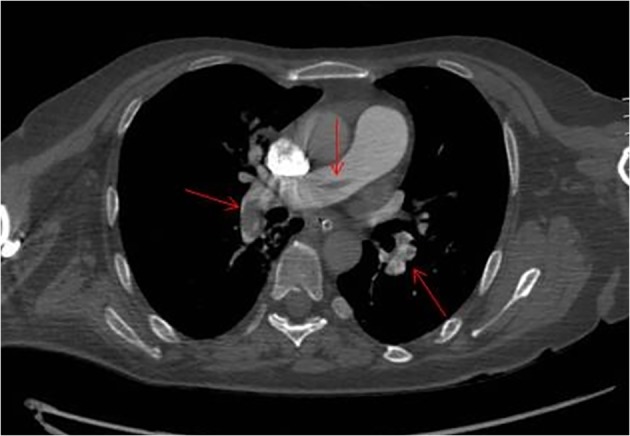
Pulmonary CT angiography axial showing multiple filling defects due to pulmonary artery embolism.

In advanced cardiovascular life support, we treated a severe hypotension with both norepinephrine and dobutamine, climbing the dobutamine by 6 to 3 µg/kg/min and increasing norepinephrine up to 0.14 µg/kg/min. In this phase, the Doppler echocardiography showed a clear right atrial dilatation. Which was the cause of PEA? The pulmonary CT angiography showed a massive bilateral PE (Fig. 1). In view of the recent and challenging surgery and the very low levels of hemoglobin (6.7 g/dL) in association with the loss of approximately 700 mL of frankly blood material from surgical drains in 24 h, it was decided not to practice thrombolysis. We organized the transfer in a highly specialized structure to operate the percutaneous fragmentation of the embolus. We started treatment with unfractionated heparin IV bolus of 80 IU/kg followed by an infusion of 18 IU/kg/h with dose adjustments according to a-prothrombin time. We simultaneously started the anticoagulant therapy with warfarin. The response to therapy was surprising. In about 3 h, the pressure came back to acceptable values and the peripheral perfusion improved. Consequently, we desisted from making the planned percutaneous fragmentation.

**Figure 2:**
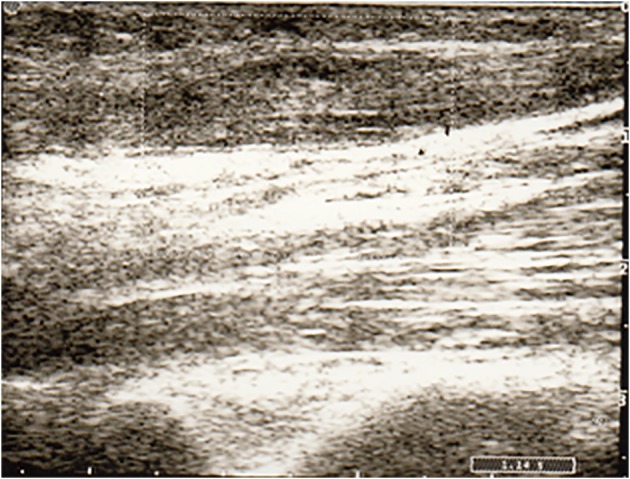
Color-Doppler scan of the right arm showing an enlarged cephalic vein, with echoic material in the lumen and absence of flow signals.

Twenty-four hours later, the breath was only slightly tachypneic, there was still hypoxia but it had significant improvement (PaO_2_/FiO_2_ indicative of moderate hypoxemia). Because the hemodynamic status was characterized by normal pressure values with slightly tachycardia, norepinephrine was gradually climbed. Meanwhile, the arm of the infusion (side cannulation of a superficial vein with a 16 Gouge catheter) and seat of the non-invasive monitoring of blood pressure (right arm) became swollen and painful. The subsequent color-Doppler ultrasound showed an extensive thrombosis of the right cephalic vein with absence of flow signals (Fig. 2). The thrombus did not extend to the axillary vein. Deep veins of the upper limbs, as well as the veins of the lower limbs, were all patent, without any sign of UEDVT or lower extremity deep venous thrombosis (LEDVT). We have researched additional conditions predisposing to thrombosis but there were no abnormality in the levels of homocysteine, as well as protein C and protein S deficiency.

Ten days after the acute episode, a new color-Doppler survey confirmed the thrombotic obliteration of the right cephalic vein with the presence of flow and patency in both the deep veins of the arm as well as the deep veins of the lower limbs. The arm became clinically deflated with clear signs of thrombophlebitis, with very hard cords along the course of the superficial veins.

## Discussion

SVT is characterized by the combination of thrombosis and inflammation in a superficial vein. It involves the great saphenous vein in more than 60-80% of cases, the small saphenous vein in 10-20% of cases and much less frequently the veins of the upper limbs. It seems to be a higher prevalence in women and its incidence increases with age in both sexes [6]. Varicose veins are the most common predisposing factor, but there is a wide range of conditions that have been outlined, such as the prolonged immobilization, trauma, obesity, and thrombophilic abnormalities of hemostasis, oral contraceptive or hormonal therapy, the previous history of LEDVT, UEDVT or SVT, the use of an intravenous catheter, malignancies and autoimmune disorders [7]. The pathophysiology of SVT can be explained in terms of external trauma, direct endothelial trauma, internal inflammation of the vein wall and alterations in hemostasis. While the external trauma can result from a direct external force (e.g. for compression by externally applied dressings), the internal trauma involves a direct endothelial damage (e.g. for a lesion by venous cannulation or for a continuous IV infusion). In both cases, the superficial vein exposed to injury produces edema and activation of leukocytes that predispose to thrombosis [8]. Thrombophlebitis is the most frequent complication of peripheral venous infusion and its most important predictor is the duration of catheterization [9]; the material and the dimensions of the catheter can affect the risk. According to Campbell [10] compared to small caliber catheters, the large bore catheters are associated with an increased risk, and the polyurethane catheters (PEU) have been associated with a reduction of 30-45% of the incidence of peripheral vein infusion thrombophlebitis compared to tetrafluoroethylene-hexafluoropropylene (Teflon) catheters. The characteristics of the solutions for intravenous administration affect the occurrence of thrombophlebitis: high osmolality solutions and low pH, such as glucose, conferring a higher risk [11]. Also, some drugs administered intravenously, such as potassium chloride, barbiturates, phenytoin, many chemotherapeutic agents and certain antibiotics (e.g. vancomycin, amphotericin B, and b-lactam antibiotics), have been associated with a doubled risk [12]. Another risk factor for SVT is the catheter infection: a percentage between 5% and 25% of peripheral catheters is colonized at the time of removal from the skin and the organisms colonized catheters are six times more likely to be associated with thrombophlebitis [13].

It remains to clarify the pathogenetic chain of events which in our case led to the considerable extension of the peripheral venous thrombosis and the massive pulmonary involvement. Probably it was the result of a synergy with enhancement of multiple risk factors: female gender, risk factors related catheter, intermittent compression of the cuff for measuring blood pressure. These risk factors have impacted the cephalic vein which is of small caliber, tortuous and full of valves and a system to 90° in the axillary vein. Not surprisingly, the axillary vein is not the first choice for placement of PICCs and Medline.

An important consideration on the pathogenesis of thromboembolism is that the case report concerns a cancer patient and then in itself already at high risk of thrombosis. It is well known that the cancer diseases increase the risk of venous thromboembolism (VTE) [14-17] and about 20% of cancer patients undergo thromboembolic events [16]. PE is the most common cause of postoperative mortality in cancer patients and is among the leading causes of death in patients with cancer [17]. In addition, gynecological surgery, even in the absence of malignancy, results in increased risk of VTE than the general surgery. Because our patient underwent to a complex surgery for ovarian cancer, she meet a higher risk for VTE.

Compression ultrasound and color-Doppler ultrasound allow detecting the venous thrombosis to assess its extent and to monitorize the thrombus evolution during the therapy [18, 19]. Nowadays, ultrasound represents the standard imaging modality and there is no diagnostic role left for phlebography [4]. Since the lower limbs represent the most frequent source of PE, usually lower extremity veins represent the first anatomical area to be investigated. In case of negative findings, the ultrasound examination is extended to the abdominal and pelvic veins as well as to the upper limbs veins. In our case, the clinical findings at level of the right upper limb, which became swollen and warm, directly drove our attention to the upper extremities.

Because data suggest that asymptomatic PE occurs up to a third of patients with DVT [20], we must assume that asymptomatic PE can occur even combined with SVT. Verlato et al [21] demonstrated a high rate of PE in patients with thrombophlebitis of the greater saphenous vein.

For these reasons, it is mandatory to stress prophylaxis of SVT in high-risk patients, such as those with cancer undergoing surgery. Prophylaxis of thrombosis is recommended for all patients admitted in oncology, with particular attention to cancer patients at high risk of thrombosis and those submitted or to be submitted to surgery [22]. Much attention should be paid to specific risk factors such as the infusion of solutions and drugs potentially harmful for the endothelium, as well as for the correct management of venous catheterization. About the changing of the peripheral catheter, a recent review found no evidence to support changing catheters every 72 - 96 h, suggesting that the insertion site should be inspected at each shift change and the catheter removed if signs of inflammation, infiltration, or blockage are present [23].

Another feature of our case was its rare as well as severe clinical manifestation: the SVT of the upper limbs became more complicated with a clinical picture of massive PE, best defined as PE with hemodynamic instability. This is a very rare report; in 1990 Sassu et al [4] reported a case of a patient with recurrent superficial thrombophlebitis of the left arm that developed right-sided PE, but not massive PE. Although Barros et al [5] described a case of post-trauma superficial thrombophlebitis of the basilic vein complicated with PE, also in this case, the PE was not massive, but limited in the basal posterior and lateral segments of the right inferior lobe.

### Conclusion

Clinicians are especially careful to prevention of embolism from LEDVT, while they often underestimated the UEDVT and SVT of the upper limb. Thanks to advances in the understanding of the pathophysiology of the SVT and the chance to make an early diagnosis, this complication although rare should be appropriately prevented, especially in cancer patients at high risk thrombosis, and its diagnosis for suspected serious complications can result. Our case is an example of the summation of numerous thrombotic risk factors simultaneously present, triggering a cascade of reactions and resulting in a potential fatal clinical manifestation.
